# Dermoscopy of pityriasis lichenoides et varioliformis acuta (PLEVA)^☆^

**DOI:** 10.1016/j.abd.2022.04.017

**Published:** 2023-09-01

**Authors:** Camilo Arias-Rodriguez, Juan Guillermo Hoyos-Gaviria, Ana María Muñoz-Monsalve, Alejandro Hernandez-Martinez

**Affiliations:** a Department of Dermatology, Universidad Pontificia Bolivariana, Medellin, Colombia; b Department of Dermatology, Specialized Diagnostic Clinic VID, Medellin, Colombia; c Department of Dermatology, Aurora Specialized Center for Skin Cancer, Medellin, Colombia; d Faculty of Medicine, Universidad Pontificia Bolivariana, Medellín, Colombia

A 24-year-old woman without significant past medical history, presented with a 12-month history of pruriginous erythematous-violaceous desquamative papules and vesicles, which appeared in crops, initially on her thighs and legs, with latter involvement of arms, forearms, and torso. Some lesions suffered central necrosis, and disappeared from 2 to 4 weeks, leaving depressed scars, with the continuous development of new crops. She referred a vitamin B-complex intramuscular injection 2 weeks preceding the start of the clinical picture, and 6 months previously she had started consuming combined oral contraceptives.

Upon clinical examination she presented several erythematous-violaceous small papules, some with a necrotic central crust, and multiple varioliform scars, with involvement of lower extremities, proximal upper extremities, chest, and abdomen (Fig. 1). A skin biopsy was performed, which showed typical findings of PLEVA, confirming the diagnosis (Fig. 2).

**Figure 1:**
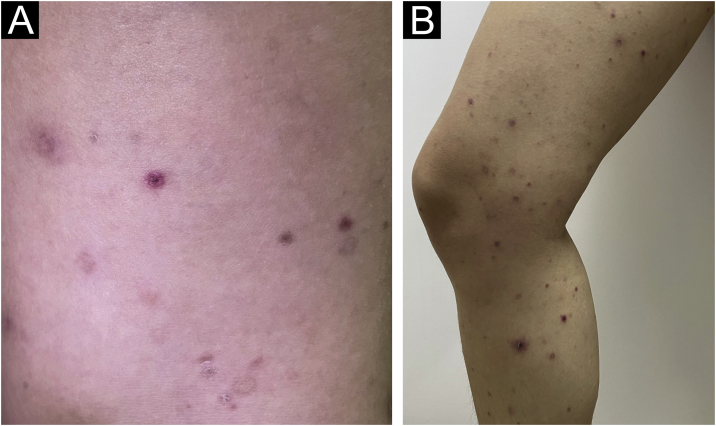
(A and B) Multiple erythematous-violaceous papules, some of them with a central necrotic crust, involving the left thigh and leg

**Figure 2:**
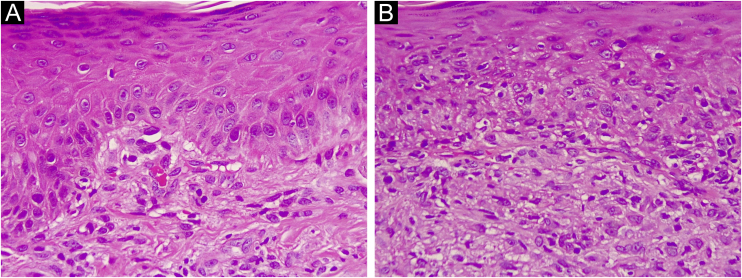
(A and B) Histopathological findings of PLEVA show orthokeratotic hyperkeratosis, mild spongiosis, vacuolar damage, lymphocyte exocytosis and subepidermal fibrin deposits, over an inflammatory infiltrate in the papillary dermis (H&E ×400)

A dermoscopic evaluation of 14 active lesions was made, with contact and no-contact polarized light dermoscopy. Vessels were found in 100% of them, with diverse morphologies (Fig. 3): all the evaluated lesions had dotted vessels, and some had linear irregular and/or glomerular vessels. Non-blanchable reddish globules were not detected. Combination of these morphologies was frequent, polymorphous vessels were present in 79% of the lesions, with dotted and irregular linear vessels being the most common combination. Vessel arrangement was peripherical in most of the lesions, although some showed uniform, clustered, or central distribution. Scales were found in most of them, white in color, with variable arrangement: the majority showed a focal location disposed in a ring-like or targetoid fashion (43%), being located between a central red-brownish clod, and a peripheral ring of vascular structures immersed in a background which generally was pink or purple, and less frequently orange or salmon-colored.

**Figure 3:**
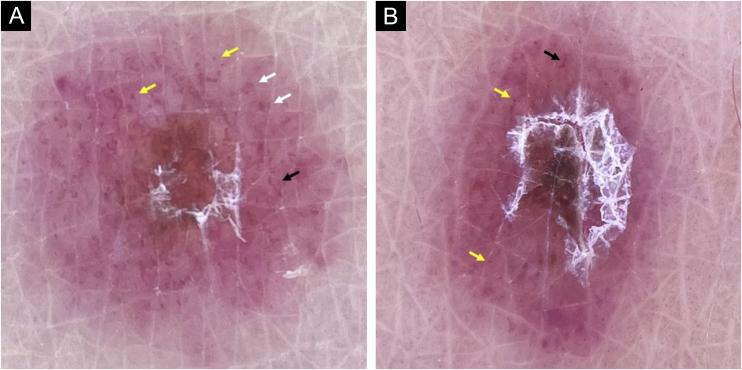
(A and B) Dermoscopy of PLEVA. Vascular morphology. Dotted vessels (yellow arrows), glomerular vessels (white arrows), and linear irregular vessels (black arrows) can be found. The most common combinations were these three types of vessels together

Two main patterns were identified: a typical target pattern, where the disposition of structures gave an image that resembled a typical target or iris lesion of erythema multiforme, consisting of three concentrical zones: central clod, intermediate ring of white scale, and peripheral vascular ring (Fig. 4). An atypical target pattern was found in other lesions, which had only two concentrical zones: a central clod, white scale or structureless area, surrounded by a vascular ring (Fig. 5).

**Figure 4:**
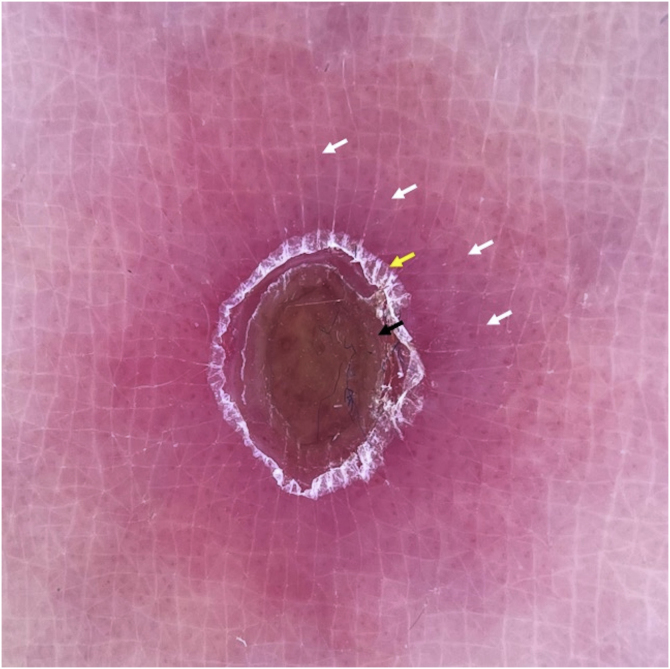
Dermoscopy of PLEVA. Typical target pattern. Polarized contact dermoscopy reveals central red-brownish clod (black arrow), intermediate ring-like focal white scale (yellow arrow) and peripheral vascular ring with a polymorphous morphology (white arrows), consisting in dotted, lineal irregular and glomerular vessels, within a pink-purplish background. These three concentrical zones give an image of a typical target

**Figure 5:**
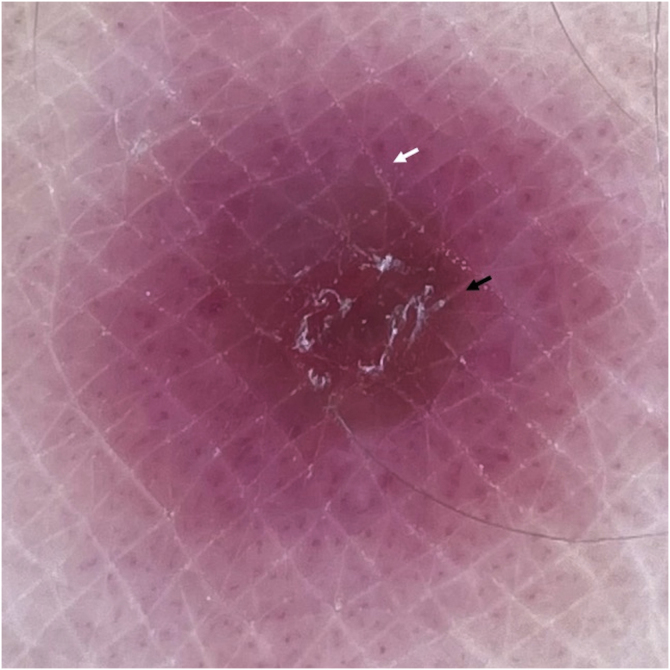
Dermoscopy of PLEVA. Atypical target pattern. Red structureless area and subtle white central scale (black arrow) surrounded by a peripheral vascular ring with polymorphous vessels and purplish background (white arrow)

Additionally, two varioliform scars were evaluated with dermoscopy, both showing light brown peripheral structureless areas with a central hypopigmented structureless area, also with a targetoid appearance, but without any vascular structures nor scales.

Pityriasis lichenoides refers to a spectrum of disease that includes three main variants: Pityriasis Lichenoides et Varioliformis Acuta (PLEVA), Pityriasis Lichenoides Chronica (PLC) and febrile ulceronecrotic Mucha-Habermann disease.^1^ As a spectrum, these conditions may sometimes overlap even though they usually have distinct clinical, histological, and dermoscopic features.^2^ PLEVA usually presents as asymptomatic erythematous macules that evolve into polymorphous erythematous papules with a necrotic center, and then disappear, leaving varioliform scars and dyschromic areas.^1^, ^3^

On dermoscopy, some authors have described a well-defined peripheral ring of vessels with a targetoid appearance, with polymorphous vessels, mainly dotted, glomerular, and/or linear irregular, as were seen in the patient. Ankad et al.^3^ proposed that these findings may correlate to blood vessels dilation and microhemorrhages in the papillary dermis.^1^, ^2^, ^4^ In two of five articles that reviewed dermoscopy of PLEVA, the targetoid pattern of vessels was not observed, though in one of them both peripheral dotted and glomerular vessels were reported.^4^, ^5^ Other authors have described non-blanchable reddish globules, which were not identified in the present case.^4^

The finding of a whitish structure was consistent in the literature, but both its morphology and arrangement varied: some described a central crust or patch while others reported a rim of scale or both.^3^, ^4^, ^5^ White structureless areas have also been reported.^4^, ^5^ Brownish central clods appear to be common, corresponding to crusted lesions, and less frequently non-blanchable reddish globules can be seen.^1^, ^3^, ^4^ In the studied patient the authors frequently found a red-brownish central cloud, surrounded by a white rim-like scale, finally encircled by a peripheral vascular ring.

Two articles depict focal blue-gray areas that may fit with scratching on the skin of color, with associated red dots.^4^, ^5^ Clarey et al.^2^ described dark and light brown dots within thin red papules, which may correspond to melanin deposition in the stratum corneum; this finding is more common in PLC.^2^ The authors did not find either of these in the presented case.

Even though dermoscopic findings of PLEVA may be diverse, they appear to be consistent, with common structures reported in literature, which were also found in the present patient. However, one feature there is no consensus about is the targetoid appearance, some authors not even describing it. The authors observed a targetoid pattern in most of the patient’s lesions, thus, for a better understanding, we propose two variants of this pattern: a typical target, with three clearly delimited concentrical zones, and an atypical target, where only two zones are present. Both could represent different stages of the disease, however, more studies in this field are required to confirm this hypothesis. Due to literature scarcity on the matter, based on the case report and previous articles, targetoid pattern could be considered a dermoscopic sign of PLEVA, especially when displaying a polymorphous vascular pattern.

## Financial support

None declared.

## Authors' contributions

Camilo Arias-Rodriguez: Study concept and design; data collection, analysis and interpretation; writing of the manuscript; critical review of the literature; final approval of the final version of the manuscript.

Juan Guillermo Hoyos-Gaviria and Ana Maria Muñoz-Monsalve: Study concept and design; effective participation in the research guidance; critical review of important intellectual content; final approval of the final version of the manuscript.

Alejandro Hernandez-Martinez: Study concept and design; data analysis and interpretation; writing of the manuscript; critical review of the literature; final approval of the final version of the manuscript.

## Conflicts of interest

None declared.
